# Hydrologically driven ecosystem processes determine the distribution and persistence of ecosystem-specialist predators under climate change

**DOI:** 10.1038/ncomms8851

**Published:** 2015-07-31

**Authors:** Matthew J. Carroll, Andreas Heinemeyer, James W. Pearce-Higgins, Peter Dennis, Chris West, Joseph Holden, Zoe E. Wallage, Chris D. Thomas

**Affiliations:** 1 RSPB Centre for Conservation Science, Royal Society for the Protection of Birds, The Lodge, Sandy, Bedfordshire SG19 2DL, UK; 2 Department of Biology, University of York, Wentworth Way, York YO10 5DD, UK; 3 Department of Environment, Stockholm Environment Institute, University of York, York YO10 5DD, UK; 4 British Trust for Ornithology, The Nunnery, Thetford, Norfolk IP24 2PU, UK; 5 Institute of Biological, Environmental and Rural Sciences, Aberystwyth University, Cledwyn Building, Penglais Campus, Ceredigion SY23 3DD, UK; 6 water@leeds, School of Geography, University of Leeds, Leeds LS2 9JT, UK; 7 ^7^water@leeds, School of Earth and Environment, University of Leeds, Leeds LS2 9JT, UK

## Abstract

Climate change has the capacity to alter physical and biological ecosystem processes, jeopardizing the survival of associated species. This is a particular concern in cool, wet northern peatlands that could experience warmer, drier conditions. Here we show that climate, ecosystem processes and food chains combine to influence the population performance of species in British blanket bogs. Our peatland process model accurately predicts water-table depth, which predicts abundance of craneflies (keystone invertebrates), which in turn predicts observed abundances and population persistence of three ecosystem-specialist bird species that feed on craneflies during the breeding season. Climate change projections suggest that falling water tables could cause 56–81% declines in cranefly abundance and, hence, 15–51% reductions in the abundances of these birds by 2051–2080. We conclude that physical (precipitation, temperature and topography), biophysical (evapotranspiration and desiccation of invertebrates) and ecological (food chains) processes combine to determine the distributions and survival of ecosystem-specialist predators.

## Abstract (web summary)

Climatic change is predicted to impact moisture-dependent ecosystems. Here Carroll *et al*. show that a combination of physical, biophysical and ecosystem processes determine the abundance and distribution of three bird species that feed on craneflies in blanket bogs.

## Graphical abstract

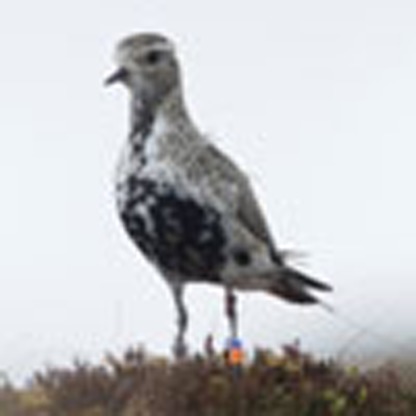

Climate change is predicted to drive substantial global biodiversity loss, with changing climatic conditions altering physical and biological ecosystem processes, in turn threatening species unable to adapt to the rapidly changing conditions^1^,^2^,^3^. This is a major concern for northern peatlands, where warmer, drier conditions under climate change could reduce ecosystem suitability for species associated with cool, wet conditions^4^,^5^. Latitudinal and elevational retreats of northern and montane species have been attributed to rising temperatures^6^,^7^, but distribution changes may also be driven by altered moisture regimes^8^,^9^, which are influenced by temperature, precipitation, soil properties, vegetation and the topography of the land^10^. However, a lack of high-resolution, landscape-scale moisture data can make moisture-driven changes harder to study. Understanding potential climate change impacts on moisture-dependent ecosystems may therefore require knowledge of the system's hydrological properties and the links between moisture and ecosystem-specialist species.

Here we consider blanket bogs, important northern and montane ecosystems defined by extensive deep peat soils over rolling terrain, which are associated with cool, wet climates^11^,^12^. An excess of precipitation over evapotranspiration, poor drainage and persistent high water tables are essential for blanket bog formation and maintenance, with water tables typically staying within 10 cm of the surface for >80% of the year^13^,^14^. They are important carbon repositories, storing over 2,000 TgC in the United Kingdom alone^15^, which contains around 18,000 km^2^ of deep peat (around 7% of the UK's land area)^11^, corresponding to 7–13% of the world's blanket bog area^16^. UK blanket bogs support a rich soil fauna^17^ and unique breeding bird and plant assemblages^18^. Craneflies (Diptera: Tipulidae) are keystone insects in blanket bog ecosystems, dominating the soil fauna's abundance and biomass^17^. Craneflies can constitute a major dietary component for peatland breeding birds (36 and 42% dry weight across studies for golden plover and dunlin, respectively)^19^; adults and chicks take both larval and adult craneflies^20^, and higher availability of adult craneflies is associated with higher chick growth and survival^21^,^22^. While habitat differences can cause spatial variation in diet composition^22^, craneflies remain important even after location effects have been accounted for, making them a key prey resource for breeding birds in British blanket bogs^19^,^20^, from the South Pennines^22^ to northern Scotland^23^. However, cranefly eggs and larvae are susceptible to desiccation, with high mortality under dry conditions^24^. As blanket bogs could experience warmer, drier conditions under climate change^5^, interactions between climate, soil moisture, invertebrates and predators may need to be identified for us to understand climate change impacts on blanket bog biodiversity.

Accordingly, we model monthly peatland water-table depth (WTD) as a function of climate and topography, and cranefly abundance as a function of WTD, to test whether hydrological processes and invertebrate food resources determine the abundances and distributions of breeding birds in blanket bogs. We validate performance of the hydrological model using data from three British blanket bogs (Moor House, Oughtershaw Moss and Lake Vyrnwy; Fig. 1), and show that both WTD fluctuations and position are well replicated. We show that modelled mean summer WTD is a significant predictor of observed adult cranefly abundance the following year, and use the resulting relationship to produce landscape-scale projections for three focal landscapes (mid Wales, South Pennines and North York Moors; Fig. 1). Within these landscapes, we show that climate change could drive large declines in cranefly abundance by 2051–2080 through changes in WTD. We then show that predicted cranefly abundance is positively related to observed abundance and persistence of golden plover, dunlin and red grouse in the South Pennines. Finally, we show that declining cranefly abundance under climate change could drive large declines in these cranefly-reliant bird species. In conclusion, we show that climate, hydrological processes and invertebrate food resources combine to determine the abundances and distributions of ecosystem-specialist predators.

## Results

### Modelling water-table depths

We used the MILLENNIA model of peatland carbon and hydrological processes^25^ to estimate blanket bog WTD as a function of simple climatic (temperature and precipitation) and topographic (slope, aspect and elevation) inputs. We updated the model to reflect monthly WTD patterns more accurately and tested predictions against dipwell observations from three British blanket bogs (Fig. 2; Supplementary Methods; Supplementary Data 1). At all sites, modelled WTD was a highly significant predictor of observed WTD, with 47–65% variation explained (Fig. 2). At Moor House and Oughtershaw Moss in northern England, where automatic dipwells sampled intact peat, mean WTD position was predicted to within 0.2 cm. At Lake Vyrnwy in mid Wales, where manual dipwells sampled peat with blocked drains, model performance declined somewhat, with a greater difference between observed and predicted WTD position and lower *R*^2^ (Fig. 2). This is to be expected, as manual monthly sampling is unlikely to accurately reflect mean WTD across the whole month. Further, local hydrology may not return to an entirely natural state after drain blocking^26^,^27^, but reduced model performance for drained peat (see also Supplementary Methods) should have little impact at landscape scales^28^. Thus, the model was able to predict WTD position and fluctuations as driven by climatic and topographic conditions.

### Modelling cranefly abundance

We hypothesized that cranefly abundance should be linked to predicted WTD, as WTD strongly influences soil moisture^29^ and cranefly eggs and larvae are sensitive to desiccation^24^. We therefore ran the hydrological model for 128 locations across three UK regions where we collected data on emerging adult cranefly abundance^4^ (Supplementary Data 2). We extracted modelled WTDs for July, August and September of the year preceding adult sampling, as conditions during early larval stages have a dominant influence on survival^24^, and hence on adult abundance^5^. Modelled WTD was a highly significant predictor of observed cranefly abundance (generalized linear model (GLM), negative binomial error and log link, with a ‘region' factor included to account for unmeasured differences among sites: intercept=5.203±0.745 s.e., *z*=6.986, *P*<0.001; estimate=−0.333±0.089, *z*=−3.766, *P*<0.001; d.f.=124, 20.3% deviance explained). Further deviance was explained by including anthropogenic drainage as an additional factor (intercept=4.885±0.681, *z*=7.178, *P*<0.001; estimate=−0.310±0.081, *z*=−3.827, *P*<0.001; d.f.=123, 39.0% deviance explained), but as parameter estimates did not differ significantly and drainage effects are highly localised^28^, we preferred the simpler model for all subsequent analyses. The proportion of deviance explained is constrained by fine-scale spatial heterogeneity in cranefly abundance^4^, but this is not expected to be relevant when aggregating density estimates at the scale of bird territories (up to 10^6^ m^2^)^22^. Hence, modelled WTD was found to predict broad-scale patterns of cranefly abundance.

The hydrological model's predictive capacity allowed us to extend WTD estimates to landscape scales. The model was run for every combination of slope, aspect and elevation in each of our three study landscapes (using a 10 × 10-m digital elevation model (DEM), and driven by observed climate data^30^ for 1914–2010). Predicted WTDs were estimated for a climatic baseline period of 1961–1990; these enabled us to estimate landscape-scale cranefly abundances using the above GLM linking WTD to cranefly abundance (Fig. 3; Supplementary Data 3). Shallow WTDs and high cranefly abundances occurred on relatively flat hilltops, and lower moisture and abundances occurred on steeper slopes, replicating observed patterns^31^. There was also an overarching longitudinal gradient, driven by geographic climate variation^4^: wettest in the west (Mid Wales; 1961–1990 mean annual rainfall, 2,071 mm; derived from UK Met Office gridded data^30^), drier in central areas (South Pennines; 1,359 mm) and driest in the east (North York Moors; 1,016 mm).

### Projecting climate change impacts

Climate change impacts were examined by driving the model with UKCP09 climate change projection data for an ‘intermediate' climate change scenario for 2011–2080 (ref. 32). Projections suggested that WTDs will become deeper over time, and hence average cranefly abundances could decline by 55.9–81.2% between the 1961–1990 baseline and 2051–2080 (Fig. 3; Supplementary Data 3). Regressions of decadal mean cranefly abundance against time indicated that all projected declines were significant (in craneflies per m^2^ per year: Mid Wales, estimate=−0.191±0.038 s.e., *R*^2^=0.648, *F*_1,14_=25.80, *P*<0.001; South Pennines, estimate=−0.121±0.038, *R*^2^=0.421, *F*_1,14_=10.17, *P*=0.007; North York Moors, estimate=−0.120±0.028, *R*^2^=0.563, *F*_1,14_=18.02, *P*<0.001; Supplementary Data 4). These declines could be conservative because the hydrological model may underestimate the deepest WTDs (Fig. 2) and there may be a moisture threshold below which craneflies cannot survive^4^. Overall, however, substantial cranefly abundance declines were projected, driven by falling summer water tables associated with warmer, drier July–September conditions.

### Effects of food availability on breeding bird populations

To quantify the extent to which craneflies influence the distribution and abundance of their predators, we examined three bird species for which craneflies are important dietary components^19^. We predicted cranefly abundance for 557 1 × 1-km grid squares in the South Pennines, where large-scale breeding bird surveys were undertaken in 1990 (ref. 33) and 2004 (ref. 34). Modelled cranefly abundance from the years immediately preceding the bird surveys (that is, 1989 and 2003) was a highly significant predictor of the abundances of all three bird species (Fig. 4; Supplementary Table 1; Supplementary Data 5), suggesting that cranefly availability can influence their abundances on a large scale.

Cranefly abundance also predicted bird population persistence: we compared predicted cranefly abundance, averaged across the two survey periods, in squares where birds were absent both years (‘empty'); present in 1990 but absent in 2004 (‘extinct'); absent in 1990 but present in 2004 (‘colonised'); or present both years (‘occupied'). Cranefly abundance differed significantly among categories for all species (Fig. 4; Kruskal–Wallis tests: dunlin *χ*^2^=102.639, d.f.=3, *P*<0.001; golden plover *χ*^2^=247.791, d.f.=3, *P*<0.001; red grouse *χ*^2^=70.834, d.f.=3, *P*<0.001). ‘Empty' squares had significantly lower cranefly abundance than other categories, and ‘occupied' squares typically had significantly higher abundance, while ‘colonised' and ‘extinct' squares were intermediate (Fig. 4; Supplementary Data 5). Hence, the highest cranefly abundances were associated with areas where bird populations appeared more stable (that is, birds were present in both survey years), while locations with intermediate abundances were seemingly less likely to sustain bird populations.

Thus, climate-driven cranefly declines would be expected to lead to reductions in dependent bird populations, particularly in the South Pennines, which is towards the southern range margin for all three focal species^35^. Projections for the South Pennines (using relationships in Fig. 4 and Supplementary Table 2) for 1990 and 2004 (bird observation periods) and 2051–2080 (under an intermediate climate change scenario) suggested that dunlin could decline by 51.1%, golden plover by 29.7% and red grouse by 14.8% (Fig. 5; Supplementary Data 6). Declining food availability could therefore drive declines in ecosystem-specialist predators, with novel extreme conditions increasing local extinction risk for dunlin and golden plover in particular.

## Discussion

Here we have shown how climate, ecosystem processes and ecological links combine to influence populations of ecosystem-specialist predators. The ability to study such processes is made possible in part because blanket bog ecosystems are strongly dependent upon high water tables^11^,^13^, and a single invertebrate group plays a very important role in the soil fauna^17^ and the diet of breeding birds^19^. Even in this relatively reductive system, however, other processes and drivers will contribute to real outcomes, meaning that unexplained variation remained for all three focal bird species. The most variation was explained for dunlin (30.5–39.4%), and the least for red grouse (8.1–8.2%). Such a pattern may be expected because, of the three focal species, dunlin have the greatest proportion of craneflies in their diet, while red grouse have the smallest^19^. Some of the remaining variation is likely to be methodological: the approach to modelling cranefly abundance may not replicate all patterns due to averaging (both across months of WTD predictions and across grid cells of cranefly predictions) and propagation of uncertainty. Further unexplained variation can be attributed to local factors that could influence bird populations, but which have not been explicitly included, such as vegetation type, land management, predation and pathogen risk, weather in wintering grounds, and density dependence^36^,^37^,^38^,^39^,^40^. It should also be noted that bird data for each grid square are based on only two survey visits in each year^41^, so some unexplained variation may be attributed to survey methods. It is notable, however, that even with such simplifications, modelled cranefly abundance still predicted up to 39% of variation in observed bird abundance, thus reflecting heavy reliance on this key food resource.

Modelled cranefly abundance was shown to differ among areas with differing bird population persistence patterns. However, peatland breeding bird abundance can show large fluctuations, with golden plover abundance at one South Pennines site fluctuating between 5 and >40 pairs over a 30-year period^5^. Cranefly populations also show interannual fluctuations, with droughts driving substantial declines and wetter conditions allowing populations to recover^4^,^24^. In a well-studied golden plover population in the South Pennines, interannual abundance fluctuations have been linked to temperature-driven fluctuations in cranefly populations^5^. Hence, even beyond the extremes of colonization and extinction, important aspects of breeding bird population dynamics may be influenced by cranefly availability. Were longer time series of bird data available, it would be beneficial to examine the degree to which bird and cranefly abundance track one another temporally and spatially, to further examine how bird population dynamics are influenced by climate-change-sensitive prey species.

On the basis of the climate change projections made here, birds breeding in blanket bogs appear to be threatened by declining food availability under climate change, driven by falling water tables. While prey switching might provide some buffering effect, alternative prey, such as Chironomidae for dunlin and Coleoptera for golden plover, are likely to show negative or neutral responses to climate change^19^. Indeed, few relevant invertebrate taxa currently found in the focal bird species' diets are likely to respond positively to increased warming or drought^19^. Further, the focal populations are towards the southern range limit for the three species^35^, so adaptations such as prey switching may be less likely to occur. Heavy reliance on craneflies, combined with the sensitivity of other potential prey to warmer, drier conditions, suggests that availability of invertebrate prey for peatland breeding birds is likely to decline under climate change.

Climate change impacts on peatland breeding bird populations will not, however, be exclusively mediated through changes in prey abundance. Golden plover might experience a mismatch between the timing of breeding and peak cranefly availability^42^, although prey abundance appears to be a stronger driver of population trends^5^. Golden plover chick growth rates are positively correlated with breeding season temperatures^43^, so higher temperatures may provide some benefits. However, breeding season weather appears to have limited population-scale impacts^40^. Further, indirect effects of climate change on vegetation structure may have a strong impact on golden plover habitat suitability^23^. For red grouse, higher temperatures before breeding are associated with earlier egg laying, larger clutch sizes and higher chick survival, but higher temperatures during breeding are associated with lower chick survival^44^. Further, another important driver of red grouse population trends, parasitism, could be affected by climate change, with higher temperatures leading to increased parasite development rates, but drier conditions potentially reducing parasite transmission^45^. For dunlin, recruitment shows a quadratic relationship with temperature, with maximum recruitment occurring at intermediate temperatures, thus possibly reflecting a balance between mortality and prey availability^46^. Climate change will undoubtedly have complex effects on breeding bird populations, integrating multiple interacting drivers^47^. However, the cranefly abundance declines projected here are likely only to have a negative effect on bird populations, and the relationships observed over recent years suggest that food availability is likely to be a dominant driver of population change^5^. Hydrological management may, therefore, be required to protect these globally important ecosystems and their associated species, perhaps focussing conservation efforts on potential population refugia where relatively high densities are predicted to remain (for example, for golden plover, Fig. 5).

Our results indicate that blanket bog WTDs predict the abundance of keystone invertebrates, and estimated invertebrate abundance predicts the abundance and dynamics of breeding birds. The links stem from physical inputs (precipitation, temperature and topography) that drive soil moisture through biophysical processes (peat development and evapotranspiration), which in turn influence invertebrate survival. We find that climate change could drive substantial declines in abundances of keystone invertebrates and their predators, acting through soil moisture. This mechanistic approach has been able to identify the consequences of climate change for ecosystem function and predators at a landscape scale. Such an approach may be useful in other ecosystems, as climate change impacts on higher-level consumers are likely to be mediated through altered species interactions^2^; comparable studies are therefore needed in other threatened ecosystems.

## Methods

### Modelling blanket bog water-table depths

The MILLENNIA peatland cohort model^25^ predicts annual peat accumulation for blanket bogs based on established relationships between climate, net primary productivity and plant litter decomposition, with plant functional types influencing litter quality and hence peat growth. Plant functional types and decomposition rates are influenced by a dynamic WTD submodel. After a spin-up period to allow the peat column to develop, the model estimates carbon stocks and fluxes (for example, peat growth) and WTD. Model inputs are the topography of the point to be modelled (that is, slope, aspect and elevation) and simple climatic inputs (mean temperature and total precipitation). The WTD submodel considers water inputs from precipitation, water loss through runoff and evapotranspiration, and the pore space available in the peat. Runoff of incoming precipitation is influenced by the topographic slope and the antecedent WTD. Water loss through evapotranspiration is influenced by plant functional types (which are determined by long-term average WTD), temperature (which is affected by aspect and elevation) and rooting depth. A model comparison study^48^ has shown that the model is suitable for modelling blanket bog conditions, producing predictions similar to those from a process-based model requiring more comprehensive local parameterization.

Here, the published model^25^, which was originally intended to produce estimates at an annual time scale, was updated to improve the representation of monthly water balances (Supplementary Methods). To evaluate performance, the updated model was driven by observed climate data, and resulting WTD predictions were tested against observed WTDs recorded from dipwells monitoring three UK blanket bogs (Supplementary Data 1). The primary evaluation used 12 years of WTD data from the Moor House Environmental Change Network site^49^ in northern England, with the model driven by climate data from a weather station at the same site. Moor House WTD data were taken from a single automatic dipwell, so to correct for systematic bias were calibrated against the average WTD from weekly readings of five nearby manual dipwells; this resulted in a −2.8-cm correction (that is, 2.8-cm shallower) applied to all months. Further evaluation was carried out using 18 months of automated dipwell data from Oughtershaw Moss in northern England and 3 years of manual dipwell data from Lake Vyrnwy in mid Wales^26^, with the model driven by UK Met Office gridded climate data^30^. Topographic data were derived from NEXTMap DEMs at a 10 × 10-m scale (Intermap Technologies. NEXTMap Britain: Digital terrain mapping of the UK. NERC Earth Observation Data Centre, 2007. Available at http://badc.nerc.ac.uk); elevation was derived directly from DEMs, while slope and aspect were calculated using standard functions in ArcMap 9.3 (ESRI; Redlands, California, USA). Model performance was assessed by comparing mean predicted and observed WTD over the evaluation period, and then by regressing observed WTD against predicted WTD (Fig. 2). Model performance was found to be highest in intact peat, with local anthropogenic drainage appearing to reduce performance (see Supplementary Methods). Overall, model performance was deemed adequate to predict both the position and seasonal fluctuations of WTDs, two key elements required to understand impacts of climate change on blanket bog hydrology.

### Modelling relationships between craneflies and WTD

**Figure 1:**
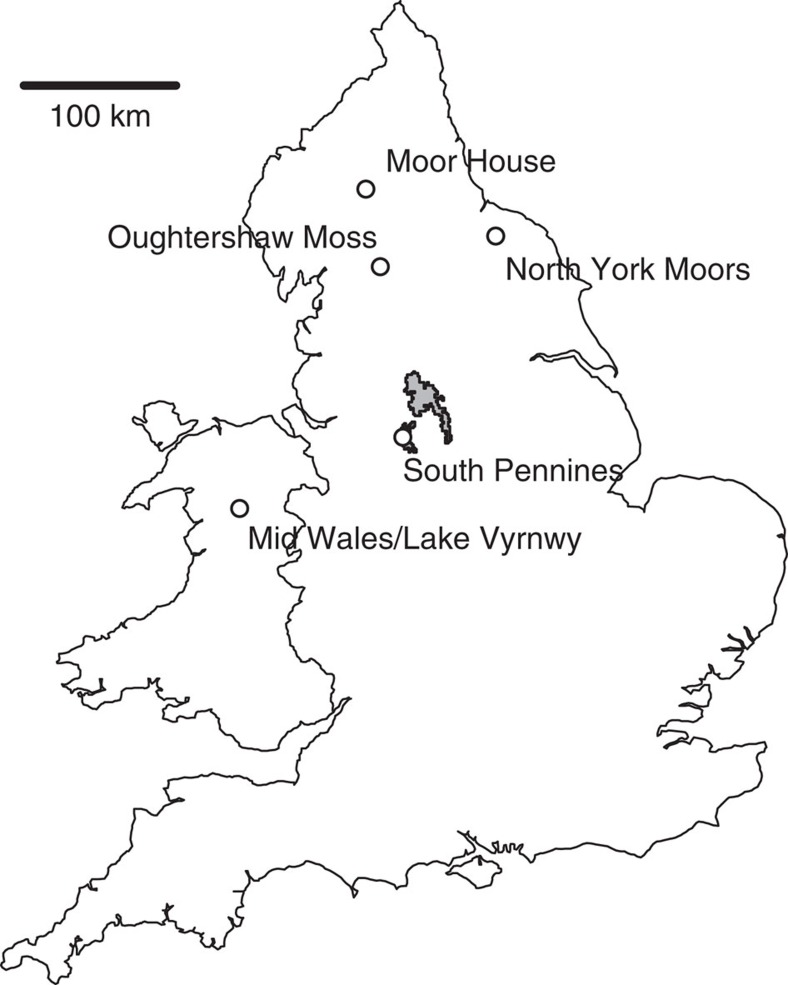
Map indicating locations of blanket bogs used in the analysis. Moor House, Oughtershaw Moss and Lake Vyrnwy indicate locations from which WTD observations were acquired to validate the hydrological model. Mid Wales, North York Moors and South Pennines indicate locations of focal landscapes where adult cranefly abundances were sampled and for which landscape-scale cranefly projections were made. Shaded area in South Pennines indicates area for which analyses of breeding birds were carried out.

**Figure 2:**
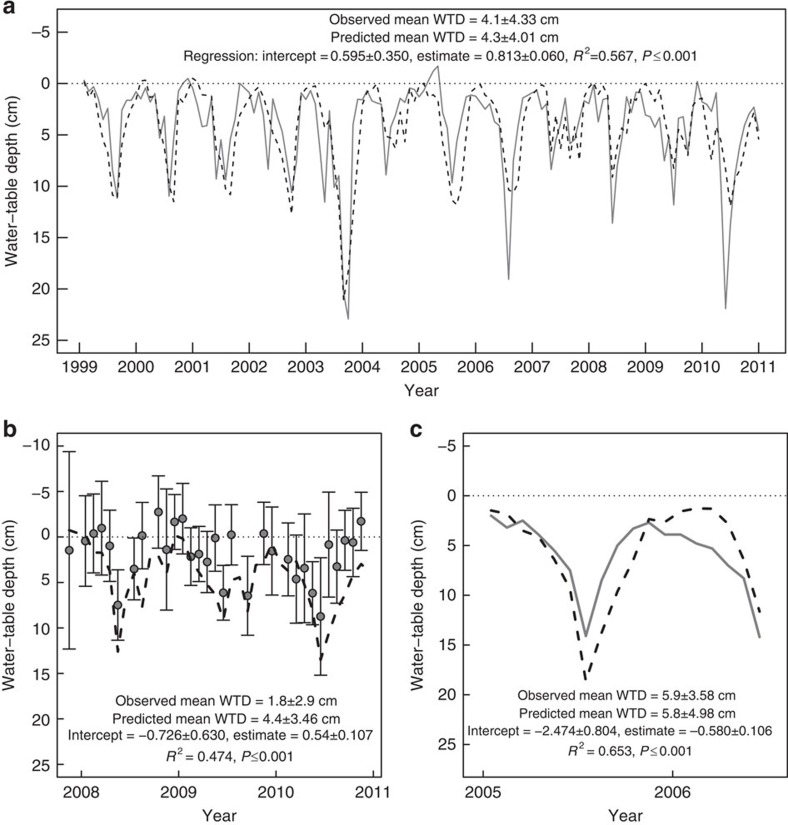
Modelled and observed monthly water-table depths for three British blanket bog sites used in MILLENNIA validation. (**a**) Moor House, northern England: observed data based on averaging hourly automated dipwell readings over 12 years (*n*=144 months); dashed black line indicates predictions, solid grey line indicates observations. (**b**) Lake Vyrnwy, mid Wales: observed data based on averaging readings from 24 dipwells sampled manually, monthly or fortnightly (*n*=31 months); dashed black line indicates predictions, points indicate monthly mean WTD, error bars indicate±s.d. of observed WTD; points plotted instead of line to reflect discrete nature of observations. (**c**) Oughtershaw Moss, northern England: observed data based on averaging readings from nine automated dipwells sampling every 20 min (*n*=18 months); dashed black line indicates predictions, solid grey line indicates observations. On all panels, 0 cm indicates the peat surface (dotted line); positive WTD values indicate a water table below the surface; negative values indicate surface ponding. Graphs all show observed and predicted mean WTD position (±s.d.) and results of regression of observed against predicted WTD, giving estimates (±s.e.), *R*^2^ and the *P* value.

**Figure 3:**
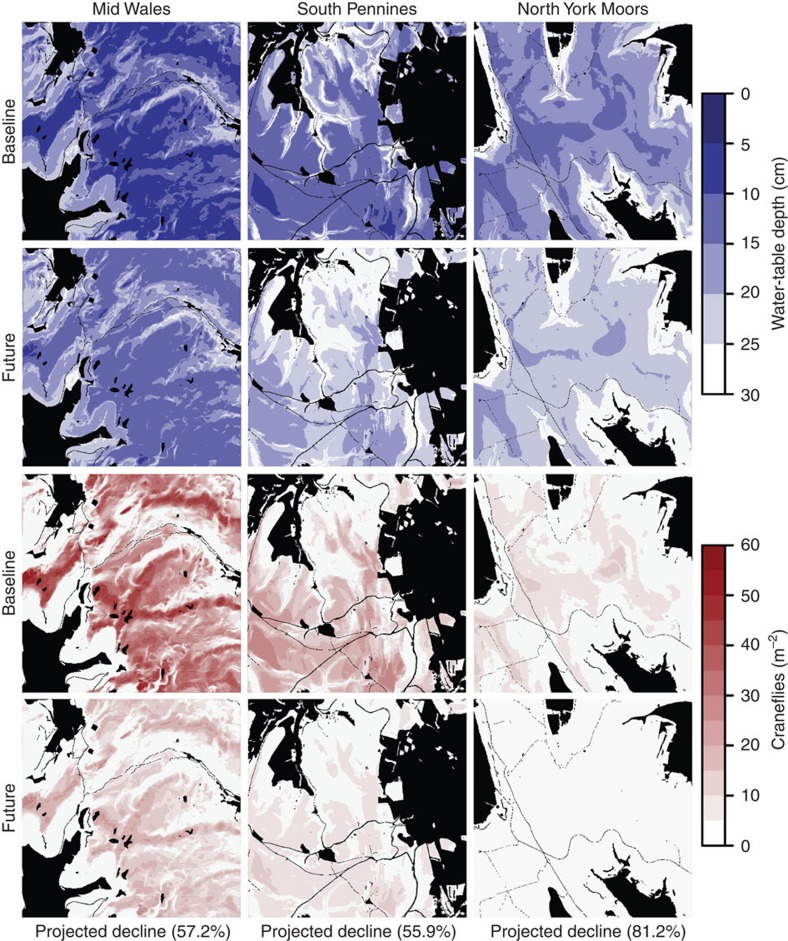
Landscape-scale projections of summer water-table depth and spring-emerging adult cranefly abundance for baseline (1961–1990) and future (2051–2080) periods. Maps show a 5 × 5-km square for each of the three focal landscapes, at 10 × 10-m resolution. Darker blue indicates shallower water tables (that is, wetter); darker red indicates higher cranefly abundance; black indicates areas unsuitable for projections (that is, non-peatland land cover, elevations below 250 m above sea level). All projections show deeper water tables and lower cranefly abundances in the future scenario. Landscapes are presented along a gradient of wettest (Mid Wales) to driest (North York Moors).

**Figure 4:**
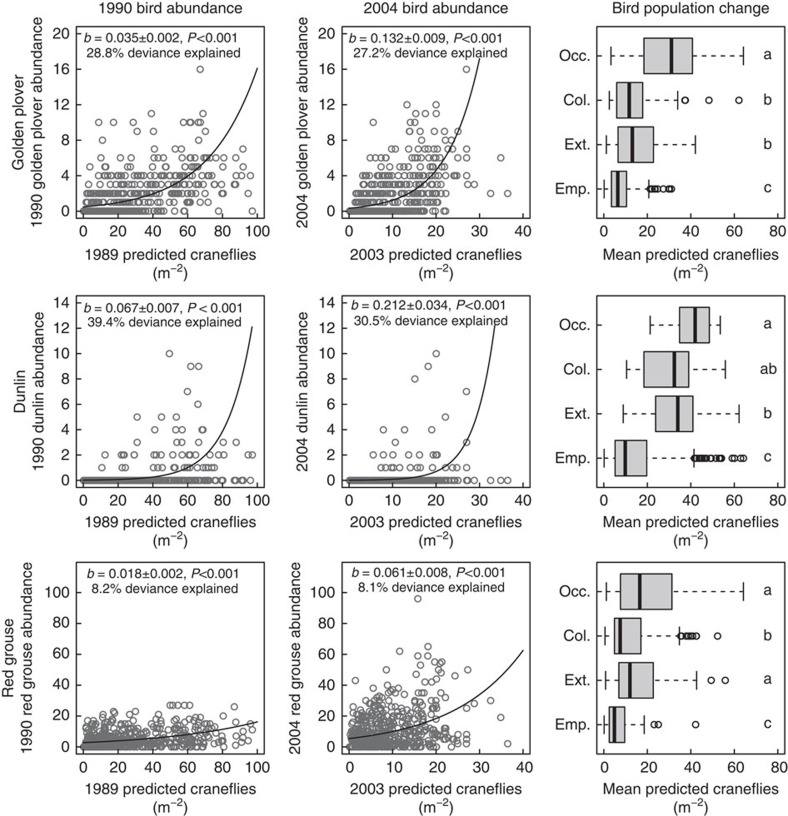
Relationships between modelled cranefly abundance and observed bird populations. First two columns show relationships between cranefly and bird abundances for 1990 and 2004 surveys; points represent values from 1 × 1-km survey squares; lines and parameter estimates are from fitted GLMs describing observed bird abundance as a function of modelled cranefly abundance. Third column shows modelled cranefly abundance for survey squares with different bird population trends: Emp., ‘empty' (birds absent both the years); Ext., ‘extinct' (birds present in 1990, absent in 2004); Col., ‘colonised' (birds absent in 1990, present in 2004); and Occ., ‘occupied' (birds present both the years). Boxes indicate interquartile range and median; whiskers indicate 1.5 × interquartile range; points indicate outliers; letters indicate results of *post hoc* Wilcoxon rank-sum tests, with different letters indicating differences significant at *P*<0.05.

To examine the relationship between cranefly abundance and WTD, observed cranefly abundance was regressed against modelled WTD. Emerging adult cranefly abundance was observed in the field in 2009 and 2010 in three British blanket bogs, using emergence traps in a large-scale replicated experimental design^4^. Sampling sites were set along a climatic gradient from mid Wales in the relatively wet west, through the South Pennines, to the North York Moors in the relatively dry east^4^ (Fig. 1). At each site, traps were active across the cranefly emergence period (late April to early July), with individual samples taken approximately every 20 days. Final abundance estimates were produced by summing counts across the entire period. Traps were set in quartets covering ∼5 × 10 m, but each trap individually sampled only around ∼0.11 m^2^. As WTD predictions were limited to the spatial resolution of the DEMs (that is, 10 × 10 m), cranefly counts from individual traps were summed to give a single abundance value per quartet of traps, meaning that resolutions of abundance observations and model projections were more closely matched. This aggregation meant that counts from 128 individual sample locations were available (Supplementary Data 2). MILLENNIA was then run for each location, with topographic variables derived from NEXTMap DEMs and the model driven by UK Met Office gridded climate data^30^, to produce monthly estimates of WTD.

In blanket bogs, cranefly adults are conspicuous during the mass emergence period in spring, but the majority of their lifecycle is spent in the upper layers of the peat^24^. Cranefly eggs and larvae experience high mortality when conditions become too dry, with the eggs and first two larval instars being the most susceptible^24^,^50^,^51^. In *Tipula subnodicornis*, the main large-bodied spring-emerging blanket bog cranefly species, the period with greatest desiccation risk is therefore approximately in July to September^24^. Emerging adult cranefly abundance in the spring is lower following warmer Augusts in the previous year^5^, which is likely to reflect impacts of drought on survival during this sensitive period. Therefore, WTDs were extracted for July, August and September (hereafter, ‘summer') of the year preceding sampling, and the mean WTD across the 3 months was calculated (Supplementary Data 2). Observed cranefly abundances were then modelled as a function of predicted summer WTD and a region factor (to account for unmeasured differences among sites, for example, peat structure and vegetation), using a GLM with negative binomial error and log link function, and fitted using the ‘MASS' R package^52^ in R v3.01 (ref. 53); modelled summer WTD was a significant predictor of observed adult cranefly abundance (see main text). Therefore, the resulting regression equation was used to convert modelled WTD to cranefly abundance. The minimum summer WTD was also trialled as a predictor, but while a significant relationship was still found, it described less variation in cranefly abundance than did mean WTD (intercept=2.976±0.220 s.e., *z*=13.516, *P*<0.001; estimate=−0.203±0.076, *z*=−2.671, *P*=0.008; d.f.=124, 16.0% deviance explained), so the mean was preferred for further analyses.

### Producing landscape-scale estimates

MILLENNIA produces point estimates, but landscape-scale patterns of WTD and cranefly abundance were required for the three focal landscapes (see above). To achieve this, the 5 × 5-km grid square covering each site was selected from the UK Met Office gridded weather dataset^30^, and slope, aspect and elevation were calculated for all 10 × 10 m cells using NEXTMap DEMs. MILLENNIA was run for all combinations of slope, aspect and elevation in each landscape, and each 10-m grid cell was assigned predicted WTD values from the run corresponding to its topography. To reduce the number of model runs required to describe each landscape, topographic variables were aggregated into intervals of 50 m for elevation, 10° for aspect and 1° for slope; interval sizes were chosen to allow the relationship between WTD and each variable to be adequately described. As model processes are one-dimensional, modelled grid cells do not interact with nearby cells. Two-dimensional processes such as accumulation of water in depressions and drainage from ridges^54^,^55^ therefore cannot be represented, meaning that this simplified spatial representation may not predict exact locations of wet or dry areas. However, this approach does allow the dominant topography and the degree of topographic heterogeneity within modelled blanket bogs to be taken into account, so important landscape components such as steep, dry slopes and flat, wet hilltops should still be reflected. When soil moisture recorded from cranefly sampling locations^4^ was regressed against modelled WTDs, WTD was found to be a significant predictor of moisture (linear regression: intercept=1.013±0.044, *t*=22.893, *P*<0.001; WTD=−0.031±0.006, *t*=−5.362, *P*<0.001; *F*_1,126_=28.75, *R*^2^=18.6%; Supplementary Data 2), showing deeper WTDs associated with drier peat and confirming that the model was able to reproduce spatial moisture patterns.

As MILLENNIA is parameterised for blanket peats, but each 5-km grid square contained a variety of habitat and soil types, areas for which model projections would not apply were removed. First, Ordnance Survey MasterMap data (Crown Copyright/database right 2012, an Ordnance Survey/EDINA supplied Service: License 100018355) allowed us to retain only ‘rough grassland' or ‘heath' habitat classifications, as these were the only two habitat types likely to reflect underlying peat. Next, as the peatlands of interest occur at high altitudes, any remaining land below 250 m above sea level was removed. Once these landscape-scale WTD estimates had been produced, the equation from the regression of cranefly abundance on summer WTD was used to convert WTD to 10-m spatial resolution estimates of cranefly abundance across the three landscapes (Supplementary Data 3).

### Producing climate change projections

To examine climate change impacts, MILLENNIA was driven by gridded weather observations^30^ from 1914 to 2010, then UKCP09 weather generator projections^32^ for the twenty-first century. The weather generator produces stochastic sequences of weather data based on 30-year climatic means on a 5 × 5-km scale^32^. For each focal landscape, the weather generator was run for each available 30-year climate period (2010–2039, 2020–2049, 2030–2059, 2040–2069, 2050–2079, 2060–2089 and 2070–2099) under an intermediate climate change scenario (SRES emissions scenario A1B). Daily outputs were aggregated to total monthly precipitation and mean monthly temperature. One hundred random realizations were produced from each run, so data within each 30-year period were sorted in order of mean summer rainfall, and the 50th driest run was selected to approximately represent the median. As 30-year periods overlapped but the model required a continuous sequence of climate input data, the middle 10 years from each were combined into one sequence of 70 years.

Modelled summer WTD for all years was converted to landscape-scale cranefly abundance estimates, as described above. To examine change over time, cranefly abundances were averaged across all 10 m cells (to account for local heterogeneity in emergence densities), thus providing a landscape-wide estimate of mean abundance for each year. As future projections were based on stochastic realizations, estimates for specific years were not of interest, making the climatic periods from which they were drawn the relevant periods in which to examine changes. Annual values were therefore aggregated to decadal means (Supplementary Data 4), and mean abundance was regressed against decade. To examine abundance changes spatially, individual decades would provide too many comparisons to allow meaningful interpretation, so projections were converted to 30-year means for 1961–1990 and 2051–2080, to represent a baseline and late twenty-first century period, respectively (note that as WTDs in summer of year *t* predicted abundances in year *t+*1, abundance estimates refer to 1962–1991 and 2052–2081, respectively; Supplementary Data 3).

### Examining relationships with birds

**Figure 5:**
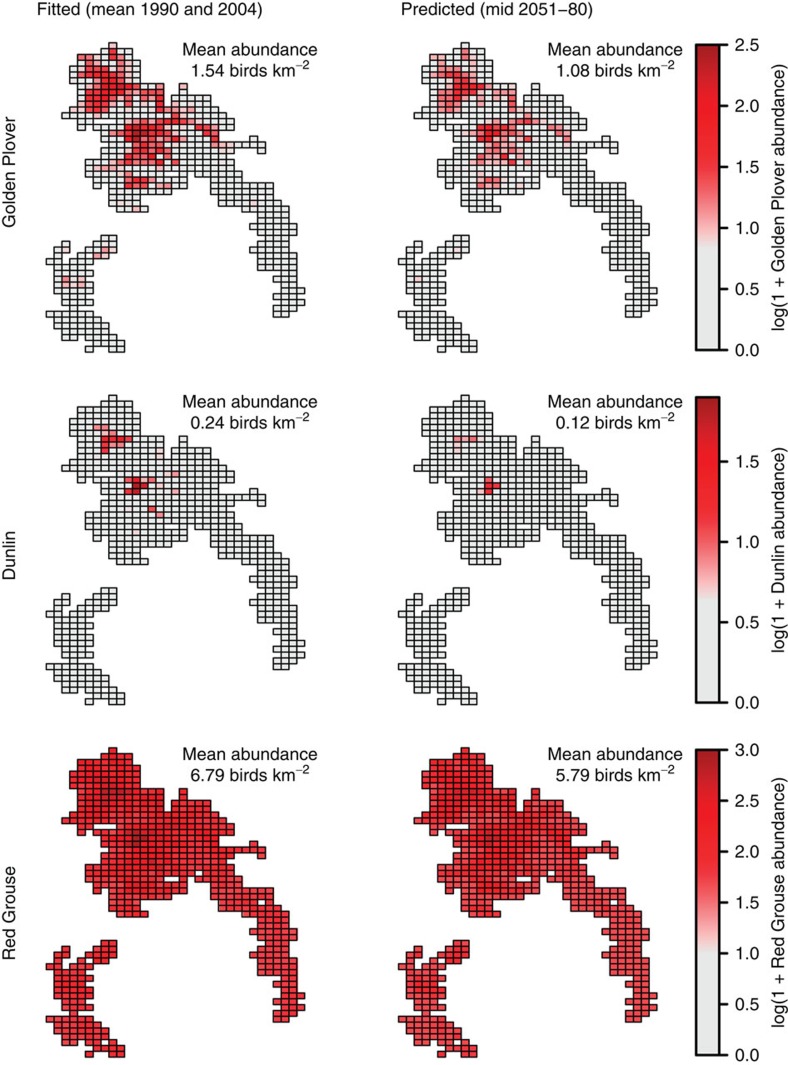
Predicted values from GLMs describing bird abundance as a function of modelled cranefly abundance. Map cells are 1 × 1-km squares with bird observations in the South Pennines, England (latitude range 53.13°–53.60° N, longitude range 2.02°–1.54° W). Fitted values are modelled abundance for years in which surveys were carried out (1990 and 2004); predicted values are those derived from applying fitted GLMs to an estimate of cranefly abundance for the middle of the 2051–2080 period.

To examine relationships between craneflies and breeding birds, data on observed bird abundances were acquired from two large-scale surveys of upland breeding birds in the South Pennines. The first survey, from 1990, was carried out by English Nature^18^ (now Natural England), and the second survey, from 2004, was conducted by Moors for the Future^19^. The 2004 survey was designed to repeat the 1990 survey to provide updated information on breeding bird distributions and abundances^19^. Data were collected for 1 × 1-km grid squares, with survey routes designed to cover a large amount of each square^19^. Two visits were made to each square during the survey period, the first between early April and mid May, and the second between mid May and late June^19^. Final counts represented the maximum number of individuals of each species observed across the two visits. For the current analysis, grid squares were restricted to those surveyed in both years, leaving 557 squares available for analysis (Fig. 1; Fig. 5).

Landscape-scale estimates of cranefly abundance were produced for all 557 grid squares by following the modelling procedure as described above. The model was again run for all combinations of slope, aspect and elevation present in the landscape, but here topographic variables were rounded into intervals of 50 m for elevation, 15° for aspect, 2° for slopes ≤20° and 5° for slopes >20°; these are larger intervals than used for the previous climate change analyses (where three 5 × 5-km grid squares were used), but as the scale of this analysis was so much greater, finer spatial resolution was less important and reducing model run time was relatively more important. The model was again driven by UK Met Office gridded weather observation data^30^. As these data corresponded to observed conditions in specific years, summer WTD estimates were extracted for 1988, 1989, 2002 and 2003 for each grid square (Supplementary Data 5). These years were chosen because abundance of adult craneflies emerging in spring of year *t* is influenced by summer conditions in year *t*−1 (refs 5, 24). However, bird abundance in year *t* (which reflects breeding success in the previous year) is likely to be influenced by cranefly abundance in year *t*−1, which would therefore be driven by summer conditions in year *t*−2 (refs 5, 24). Hence, summer conditions in 1988, 1989, 2002 and 2003 would influence emerging adult cranefly abundance in 1989, 1990, 2003 and 2004, respectively, which in turn could influence bird populations in 1990 and 2004.

Once cranefly abundance estimates had been produced, estimates were extracted for each 1 × 1-km bird survey square (averaging all 10-m grid cells within each 1-km survey square), and used as the predictor variable in models of breeding bird abundance. Three montane peatland breeding bird species, dunlin (*Calidris alpina*), golden plover (*Pluvialis apricaria*) and red grouse (*Lagopus lagopus scoticus*) were selected for this analysis given the importance of craneflies to them during the breeding season^19^. Abundances of all three focal species differed significantly from a Poisson distribution, with overdispersion appearing common, hence all species were modelled with a negative binomial error and log link function using the ‘MASS' R package^52^. Spearman correlations between the 2 years were only moderate (golden plover, *ρ*=0.579; dunlin, *ρ*=0.405; red grouse, *ρ*=0.512), so it was determined to be acceptable to analyse the 2 years separately. Relationships with modelled cranefly abundance were assessed by examining the significance of parameter estimates and the proportion of deviance explained; % deviance explained was calculated as (null deviance−residual deviance)/null deviance. In all cases, cranefly abundance from year *t*−1 (that is, driven by summer WTD in year *t*−2) described more deviance (Supplementary Table 1) so these estimates were used for further analyses; this was also the most biologically plausible model (see above).

To ensure that results were not an artefact caused by spatial autocorrelation, models were rerun in a generalized additive model (GAM) framework, fitted using the ‘mgcv' R package^56^. GAMs included a two-dimensional tensor product smooth term that used a thin-plate regression spline basis^57^, which was fitted to *x* and *y* coordinates to account for underlying spatial structure in the data. Results were similar to those from GLMs, with parameter estimates of a similar magnitude and all significant relationships remaining significant (Supplementary Table 3). Therefore, confidence was increased that results were not caused only by spatial structure in the data; accordingly, GAM results are discussed no further.

To examine relationships between modelled cranefly abundance and bird population persistence, 1-km survey squares were split into four categories based on observed abundance of each bird species (Supplementary Data 5). ‘Empty' squares had no birds in either 1990 or 2004; ‘colonised' squares had no birds in 1990, but at least one in 2004; ‘extinct' squares had at least one bird in 1990, but none in 2004; and ‘occupied' squares had birds in both years. Average cranefly abundance was calculated across the two survey years (that is, 1989 and 2003 abundance, which influenced 1990 and 2004 bird abundance, respectively); Kruskal–Wallis tests were then used to test differences in average cranefly abundance among the square categories, with pairwise differences examined using *post hoc* Wilcoxon rank-sum tests.

Finally, possible impacts of climate change on South Pennines bird populations were examined. Relationships between observed bird abundance and modelled cranefly abundance were re-fitted using data from both 1990 and 2004 in the same model to parameterise a more general relationship (Supplementary Table 2); fitted estimates of this model were used to give ‘baseline' bird abundances for the survey period. Estimates of future cranefly abundance were calculated by applying the climate change regression equation for the South Pennines (see main text) to the mean cranefly abundance from the two survey years; abundance estimates were made for 70 years in the future relative to the mean survey date (that is, the mid 1990s), thus representing the mid-point of the 2051–2080 period (that is, the mid 2060s). The regression equation was derived from only one of the 5 × 5-km grid squares to prevent stochastic, spatially incoherent runs of the UKCP09 weather generator producing artefacts at the boundaries of grid squares. Once future estimates of cranefly abundance had been produced, these were entered into the fitted GLM as new predictor values to approximate possible future bird abundances (Supplementary Data 6).

### Code availability

MILLENNIA model code as used in this study are stored on a secure server of the Stockholm Environment Institute; please contact Andreas Heinemeyer, at andreas.heinemeyer@york.ac.uk, for access.

## Additional information

**How to cite this article:** Carroll, M. J. *et al*. Hydrologically driven ecosystem processes determine the distribution and persistence of ecosystem-specialist predators under climate change. *Nat. Commun.* 6:7851 doi: 10.1038/ncomms8851 (2015).

## Supplementary Material

Supplementary Tables, Methods and References Supplementary Tables 1-3, Supplementary Methods and Supplementary References

Supplementary Data 1 Data for comparison of modelled and observed WTDs

Supplementary Data 2 Data for analysis of relationships between cranefly abundance, moisture and modelled WTD

Supplementary Data 3 Landscape-scale modelled estimates of WTD and cranefly abundance

Supplementary Data 4 Decadal mean cranefly abundance estimates for climate change projections

Supplementary Data 5 Data for analysis of relationships between modelled cranefly abundance and observed bird populations

Supplementary Data 6 Data for projections of bird abundance under climate change
