# Supplementary material for: Hydrologically driven ecosystem processes determine the distribution and persistence of ecosystem-specialist predators under climate change
Source: Nat Commun. 2015 Jul 31;6:7851. doi: 10.1038/ncomms8851 (PMC4532857; doi:10.1038/ncomms8851)
Supplement: Supplementary Tables, Methods and References — Supplementary Tables 1-3, Supplementary Methods and Supplementary References [file ncomms8851-s1.pdf]

Supplementary Table 1. Extended results from GLMs describing observed bird abundance as a function of modelled cranefly abundance. For intercept and cranefly abundance term, table shows parameter estimates (± standard error), *z* value and associated *P* value.

	Intercept	Cranefly abundance parameter estimate	DF	Deviance explained
1990 Golden Plover, 1989 craneflies	-0.733 (± 0.095)<br>z = -7.741<br>P < 0.001	0.035 (± 0.002)<br>z = 15.309<br>P < 0.001	555	28.8%
1990 Golden Plover, 1990 craneflies	-0.052 (± 0.079)<br>z = -0.664<br>P = 0.507	0.873 (± 0.088)<br>z = 9.920<br>P < 0.001	555	12.8%
2004 Golden Plover, 2003 craneflies	-1.106 (± 0.121)<br>z = -9.136<br>P < 0.001	0.132 (± 0.009)<br>z = 13.991<br>P < 0.001	555	27.2%
2004 Golden Plover, 2004 craneflies	-0.256 (± 0.090)<br>z = -2.832<br>P = 0.005	1.349 (± 0.124)<br>z = 10.878<br>P < 0.001	555	13.3%
1990 Dunlin, 1989 craneflies	-4.019 (± 0.332)<br>z = -12.114<br>P < 0.001	0.067 (± 0.007)<br>z = 9.863<br>P < 0.001	555	39.4%
1990 Dunlin, 1990 craneflies	-2.229 (± 0.211)<br>z = -10.563<br>P < 0.001	1.356 (± 0.225)<br>z = 6.031<br>P < 0.001	555	15.4%
2004 Dunlin, 2003 craneflies	-4.594 (± 0.479)<br>z = -9.580<br>P < 0.001	0.212 (± 0.034)<br>z = 6.293<br>P < 0.001	555	30.5%
2004 Dunlin, 2004 craneflies	-3.078 (± 0.316)<br>z = -9.732<br>P < 0.001	2.497 (± 0.418)<br>z = 5.971<br>P < 0.001	555	11.2%
1990 Red Grouse, 1989 craneflies	1.019 (± 0.082)<br>z = 12.383<br>P < 0.001	0.018 (± 0.002)<br>z = 7.544<br>P < 0.001	553	8.2%
1990 Red Grouse, 1990 craneflies	1.274 (± 0.066)<br>z = 19.185<br>P < 0.001	0.497 (± 0.082)<br>z = 6.075<br>P < 0.001	553	5.8%
2004 Red Grouse, 2003 craneflies	1.687 (± 0.090)<br>z = 18.736<br>P < 0.001	0.061 (± 0.008)<br>z = 7.616<br>P < 0.001	551	8.1%
2004 Red Grouse, 2004 craneflies	2.151 (± 0.068)<br>z = 31.822<br>P < 0.001	0.379 (± 0.104)<br>z = 3.639<br>P < 0.001	551	1.9%

Supplementary Table 2. Results from GLMs describing observed bird abundance in the South Pennines as a function of modelled cranefly abundance, including data from both 1990 and 2004, and including a 'year' fixed factor. For intercept and cranefly abundance term, table shows parameter estimates (± standard error), *z* value and associated *P* value. These relationships were used along with projections of cranefly abundance for the mid-point of the 2051-80 period to estimate future projected bird abundances.

	Intercept	Cranefly abundance parameter estimate	DF	Deviance explained
Golden Plover	-0.587 (± 0.071)<br>$z = -8.212$<br>$P < 0.001$	0.045 (± 0.003)<br>$z = 17.350$<br>$P < 0.001$	1111	20.8%
Dunlin	-3.873 (± 0.257)<br>$z = -15.079$<br>$P < 0.001$	0.086 (± 0.008)<br>$z = 10.587$<br>$P < 0.001$	1111	32.0%
Red Grouse	1.494 (± 0.056)<br>$z = 26.572$<br>$P < 0.001$	0.022 (± 0.002)<br>$z = 9.423$<br>$P < 0.001$	1105	12.6%

Supplementary Table 3. Results from GAMs describing observed bird abundance as a function of modelled cranefly abundance, including 2-dimensional tensor product smooth fitted to *x* and *y* coordinates to account for spatial structure in the data. For intercept and cranefly abundance term, table shows parameter estimates (± standard error), *z* value and associated *P* value. Results were similar to those from GLMs without spatial terms, so GAM results are not discussed further.

	Intercept	Cranefly abundance parameter estimate	DF	Deviance explained
1990 Golden Plover, 1989 craneflies	-0.993 (± 0.112) $z = -8.887$ $P < 0.001$	0.037 (± 0.003) $z = 12.509$ $P < 0.001$	541.151	41.6%
1990 Golden Plover, 1990 craneflies	-0.378 (± 0.094) $z = -4.013$ $P < 0.001$	0.670 (± 0.093) $z = 7.195$ $P < 0.001$	535.783	36.5%
2004 Golden Plover, 2003 craneflies	-1.187 (± 0.133) $z = -8.918$ $P < 0.001$	0.116 (± 0.011) $z = 10.835$ $P < 0.001$	544.421	38.6%
2004 Golden Plover, 2004 craneflies	-0.465 (± 0.106) $z = -4.396$ $P < 0.001$	0.779 (± 0.121) $z = 6.432$ $P < 0.001$	538.300	35.6%
1990 Dunlin, 1989 craneflies	-4.664 (± 0.576) $z = -8.091$ $P < 0.001$	0.065 (± 0.008) $z = 7.811$ $P < 0.001$	549.140	46.0%
1990 Dunlin, 1990 craneflies	-3.638 (± 0.990) $z = -3.677$ $P < 0.001$	1.208 (± 0.251) $z = 4.808$ $P < 0.001$	543.606	39.7%
2004 Dunlin, 2003 craneflies	-9.874 (± 3.972) $z = -2.486$ $P = 0.013$	0.172 (± 0.038) $z = 4.468$ $P < 0.001$	548.148	49.9%
2004 Dunlin, 2004 craneflies	-11.000 (± 5.171) $z = -2.127$ $P = 0.033$	1.032 (± 0.404) $z = 2.554$ $P = 0.011$	548.014	43.2%
1990 Red Grouse, 1989 craneflies	0.879 (± 0.089) $z = 9.904$ $P < 0.001$	0.016 (± 0.003) $z = 5.702$ $P < 0.001$	538.182	26.6%
1990 Red Grouse, 1990 craneflies	1.122 (± 0.064) $z = 17.560$ $P < 0.001$	0.389 (± 0.087) $z = 4.491$ $P < 0.001$	537.906	25.7%
2004 Red Grouse, 2003 craneflies	1.480 (± 0.083) $z = 17.800$ $P < 0.001$	0.047 (± 0.008) $z = 6.045$ $P < 0.001$	534.432	43.1%
2004 Red Grouse, 2004 craneflies	1.766 (± 0.056) $z = 31.818$ $P < 0.001$	0.389 (± 0.090) $z = 4.323$ $P < 0.001$	533.860	42.0%

Supplementary Methods: updating the MILLENNIA model to model peatland hydrology

The MILLENNIA peat cohort model[1] was developed to examine and predict long-term peatland development patterns at an annual timestep. The model could be driven with monthly data[2], but for more extensive use on this shorter timestep, model equations needed to be updated to adequately reflect sub-annual water-table behaviour. This section describes how the model was updated for predicting monthly water tables.

The fundamental modelling processes remained the same as in the annual version[1]. The model represents peat as a one-dimensional column, in which all processes occur vertically and happen instantaneously. The model starts from the approximate start of peat formation up (e.g., 10,000 years ago). Rainfall and temperature drive the dynamic water-table sub-model, which in turn influences vegetation composition, with associated feedbacks on the water table via evapotranspiration losses. The vegetation is split into above-ground and below-ground material, and into different chemical fractions[3]. Litter from vegetation is added to an annual cohort of peat, either at the surface (shoots) or throughout the peat profile (roots), which then undergoes erosion, decay and compaction over time. Carbon in the system can be lost via erosion or as a gas, with the conversion to CO_2 or CH_4 determined by the position of the water table. Therefore, over time, peat columns grow or shrink and emit gases, with these processes driven by the dynamically-varying water table and vegetation composition.

The dynamic water table model

Water-table variation is driven by input from rainfall, losses from runoff and evapotranspiration and a small, constant drainage out of the system to reflect loss into the bedrock. To drive these processes, each modelled peatland is assigned total monthly rainfall (mm) and mean monthly temperature (°C). Each column within the peatland is also assigned values for slope (°), aspect (°) and elevation (m).

Topography is used to modify weather inputs to provide locally-adjusted values for each peat column. Annual rainfall increases with elevation by 2.25 mm m^{-1}. Although this relationship varies from 0 mm m^{-1} to 4.5 mm m^{-1} around the UK [4], this value represents a compromise to make the relationship widely-applicable. Equation 1 describes local rainfall adjustment:

$$R_L = R_O + \frac{2.25}{12} * E \qquad (1)$$

Here, R_L = local rainfall, R_O = observed rainfall, and E = relative elevation, i.e., the difference in metres between the modelled location and the elevation that weather data refer to. Therefore, a negative relative elevation decreases local rainfall, while a positive relative elevation increases local rainfall. For the monthly model, the 2.25 mm m^{-1} change is divided by 12, so that when summed over the year, the annual change is 2.25 mm m^{-1}.

Temperature decreases with elevation by 0.006°C m^{-1}, which should be applicable throughout the UK [5]. Temperature is also adjusted by slope and aspect to account for variation in incident radiation [6]. The equation used in the annual model[1] is retained for the monthly model:

$$T_L = T_O - 0.006 * E + \left((\cos(S) * cos(L) + \sin(S) * \sin(L) * \cos(A - 200)) - \cos(L)\right) * 10 \quad (2)$$

Here, T_L = local temperature (°C), T_O = observed temperature (°C), E = relative elevation (m), S = local topographic slope (°), L = site latitude (°), A = local aspect (°). The strength of the slope-aspect-temperature relationship varies with latitude to reflect variation in solar angle. The use of cos(aspect-200) means that the maximum temperature occurs at 200° aspect, while the minimum occurs at 20°. On southerly aspects, temperature increases with slope, while on northerly aspects, temperature decreases with slope.

Water input occurs via precipitation; water is lost through evapotranspiration and runoff. Evapotranspiration is calculated using the Thornthwaite equation[7] as this only requires precipitation and temperature inputs, and can be used for humid and wetland areas[8]. Potential evapotranspiration (PET) estimates are adjusted based on the WTD and vegetation composition to give actual evapotranspiration (AET); AET decreases as WTD becomes deeper [9, 10]. The relationship between the AET:PET ratio and WTD changes between plant functional types (PFTs), and is estimated based on maximum rooting depths, root profile distribution and the shape of published relationships[11, 12]: declines in AET occur sooner and faster in PFTs with shorter roots. For *Sphagnum* and other bryophytes, minimum AET is 50% of PET[12]; for all other PFTs, minimum AET is 80% of PET[13]. AET:PET relationships are described in the table below. The relationship between PFT proportions and WTD is the same as in the annual model[1].

Summary of relationships between AET:PET ratio and WTD for different plant functional types used in the model.

Plant functional type	Minimum AET:PET ratio	WTD where AET starts to fall (cm)	WTD when AET reaches minimum (cm)	Shape of relationship
Sphagnum	0.5	0	~40	Approximates published shape[12]
Bryophytes (e.g., *Polytrichum commune*)	0.5	0	~40	Approximates published shape[12]
Herbs (e.g., *Potentilla erecta*)	0.8	0	~12	Linear
Shrubs (e.g., *Calluna vulgaris*)	0.8	5	~19	Linear
Grasses (e.g., *Molinia caerulea*)	0.8	10	~30	Linear
Rushes (e.g., *Juncus squarrosus*)	0.8	10	~38	Linear
Sedges (e.g., *Eriophorum vaginatum*)	0.8	10	~38	Linear

Water inputs and outputs are summed to give a change value. As in the annual model, an exponential relationship is assumed between distance to the water table and available pore space, such that available space increases with distance from the water table. Total space is calculated by integrating over the available unsaturated peat cohorts. By combining the water entering the system with the available space, a new WTD is calculated.

Monthly runoff equations

The model does not use formal hydrological runoff process functions, but instead uses equations that aim to reproduce dominant water-table behaviour observed in blanket peat. Runoff varies from 90% to <10% of rainfall[14], so equations were developed to reflect this. Runoff is strongly influenced by the existing water table, so runoff is a function of the previous time step's WTD; based on published data[14], runoff was assumed to be related to WTD exponentially, but with runoff higher and more sensitive to WTD when the water table is within 5 cm of the surface. Runoff also increases with slope angle[15, 16], so a cos function was used to increase runoff as slope steepness increases. A condition was set such that runoff could never be higher than total precipitation.

The runoff equation used is determined by WTD in the preceding time step. Hence, if the antecedent WTD is deeper than 5 cm, Equation 3 is used to calculate runoff:

$$\left(1 - \left(\alpha - \beta * (\exp(-0.01 * WTD)^{\gamma})\right) - \kappa \cos(\lambda * S)\right) * R_L \qquad (3)$$

Here, *WTD* = previous time step's WTD, *S* = slope and R_L = local rainfall; see table in section *Runoff equation parameterisation and sensitivity* for parameter descriptions and fitted values. This is based on the equation used in the annual model, and produces an exponential relationship between runoff and WTD; as the water table moves deeper, less rainfall is lost as runoff. After parameterisation (see below), at a slope of 0 the minimum runoff was 7.5% of rainfall, while at a WTD of 5 – 10 cm, runoff ranged from 60% to 50%.

When antecedent WTD is within 5 cm of the surface, runoff is calculated by Equation 4:

$$(\delta - (0.01 * WTD^2) - \kappa \cos(\lambda * S)) * R_L \qquad (4)$$

WTD, *S* and R_L are as for Equation 3; see table in section *Runoff equation parameterisation and sensitivity* for parameter descriptions and fitted values. Here, to reflect the higher, more sensitive runoff, a quadratic function of WTD is used. For the fitted model, runoff when WTD = 5 cm was 65% of rainfall, rising to 90% when WTD = 0 cm.

It was assumed that if water was standing on the surface, almost all rainfall would run off. An exponential relationship was once again used, and is shown in Equation 5:

$$\left(\varepsilon - \left(\eta * \left(\exp(0.01 * WTD)^{\theta}\right)\right) - \kappa \cos(\lambda * S)\right) * R_L \qquad (5)$$

WTD, *S* and R_L are as for Equation 3; other parameters and values used are described in table in section *Runoff equation parameterisation and sensitivity*. As runoff data from situations with standing water were not available, the fitted model was set to produce a minimum of 95% runoff, but not to vary strongly with the depth of standing water.

Stabilising modelled WTD behaviour

After runoff is calculated, total water input for the time step is calculated as precipitation – (evapotranspiration + runoff). However, individual months with relatively high or low precipitation

could cause unstable behaviour, with rapid flooding and droughts occurring more easily and frequently than observed in real blanket bogs[14, 17]. Therefore, after water input was calculated, functions were applied to stabilise model behaviour.

In months of relatively high precipitation, runoff and evapotranspiration were not always sufficient to account for incoming precipitation, causing available pore space to be rapidly used up, in turn causing rapid and long-lasting floods. This probably reflected the inability of the model to represent processes such as interception of rainfall by vegetation, increased surface runoff during high intensity rain events, or snowfall during winter months. Hence, for relatively wet sites, in months where precipitation was greater than average, a correction to water input was applied, shown in Equation 6:

$$W_C = W_U * (R_{ratio})^{-\mu} \qquad (6)$$

Here, W_C is the corrected water input, W_U is the uncorrected water input (i.e., precipitation – (evapotranspiration + runoff)), R_{ratio} is the ratio of observed monthly rainfall to that year's average monthly rainfall (i.e., annual rainfall/12), and μ is the power-law correction factor. The correction was only used when $R_{ratio} > 1$ and for sites with average annual rainfall (R_{av_ann}) > 1500 mm. Initially derived with $\mu = 1$, it was found that wetter sites were more susceptible to flooding, so μ varied with R_{av_ann}: $1500 \leq R_{av\_ann} < 1625$, $\mu = 1$; $1625 \leq R_{av\_ann} < 1750$, $\mu = 2$; $1750 \leq R_{av\_ann} < 2000$, $\mu = 3$; $2000 \leq R_{av\_ann}$, $\mu = 4$. The correction operates such that as the disparity between that month's precipitation and the mean monthly precipitation increases, water input becomes smaller, representing processes such as changes in surface runoff with rainfall intensity. The correction is applied to water input rather than runoff, because the model erosion term, which is calculated as a function of runoff, becomes too large if runoff is dramatically increased. Hence, the correction ensures that in a heavy rainfall event, areas with moderately high WTDs do not experience rapidly-occurring, long-lasting floods, but areas with lower WTDs still experience re-wetting.

Further, whilst rapid, short-term droughts under very low rainfall conditions were well-replicated, under moderately low rainfall, relatively wet sites could switch rapidly to drought conditions. This was likely either because evapotranspiration or runoff was too high under these conditions. Therefore, a further stabilisation factor was added, shown in Equation 7:

$$\begin{aligned} 0.5 \leq R_{ratio} < 0.75;\ W_C &= W_U * R_{ratio} \\ 0.25 \leq R_{ratio} < 0.5;\ W_C &= W_U * (1 - R_{ratio}) \end{aligned} \qquad (7)$$

Variable definitions are as for Equation 6. The correction reached a maximum when $R_{ratio} = 0.5$, and then decreased again as R_{ratio} approached 0.25 or 0.75; a graduated correction was used to avoid sharp transitions from uncorrected to corrected water input. Hence, the correction allowed the model to continue representing droughts in very dry conditions well, but in months with only moderately low rainfall, water tables fell more slowly. Both corrections were derived by trialling the model for the evaluation datasets (see below) and observing resulting behaviour of model predictions; correction factors were accepted when flood and drought behaviour stabilised across all evaluation datasets.

Runoff equation parameterisation and sensitivity

Parameter values were set by trialling different values and comparing model output to observed data. Values were initially chosen to approximately reproduce published runoff proportions[14] at slope = 0°. When suitable values were found, the model was run for an Environmental Change Network (ECN) site at Moor House, Cumbria, northern England, for which observed monthly WTD data were freely-available[18]; values were accepted if they predicted WTD fluctuations for 1999 – 2003 well; R^2 from a regression of observed vs. predicted WTD and measures of WTD position (mean, standard deviation, minimum, maximum) were used to judge model performance. As standing water was rarely observed, Equation 5 was parameterised by choosing values that did not cause dramatic water table drops due to runoff being too high, but did not allow standing water to last longer than observed.

A simple sensitivity analysis was carried out, where each value was raised and lowered by 10%. The range in R^2 was 0.15, but this was primarily caused by ε being lowered; excluding ε-10%, the range was only 0.06. The range in mean WTD was 2.6 cm; this variation appeared to be caused by ε-10% and κ+10%, and when these were excluded, the range was only 1.5 cm. The most sensitive value was minimum WTD, which had a range of 5.2 cm, but this was again caused by ε-10%; when this was excluded, the range was only 2.3 cm. Maximum WTD was relatively insensitive, with a range of only 1.6 cm. The mean absolute error relative to observed data was 0.6 cm for mean WTD, 0.5 cm for minimum WTD and 1.9 cm for maximum WTD, indicating relatively robust performance. Qualitatively, there was little effect on model predictions from parameter value variation; only ε-10% caused substantially different behaviour, allowing floods to occur more frequently.

The strength of the slope effect (parameter λ) could not be parameterised using the Moor House data, as data were not available over a range of slopes. To set the value, modelled peat depth (which, via erosion, is ultimately driven by runoff) was compared to observed peat depth data across shallow, moderate and steep slopes at Lake Vyrnwy (A. Heinemeyer, unpublished data). The slope parameterisation was accepted when it broadly reproduced observed patterns. The use of cos(4.5*slope) meant that no slopes over 40° could be modelled with this parameterisation.

Parameters used in monthly runoff equations and fitted values.

Parameter	Equation	Description	Value
α	3	Defines minimum runoff at slope = 0 (i.e. 1 - α - κ)	0.425
β	3	Strength of WTD effect below surface (adjusts slope of relationship)	0.7
γ	3	Exponent of WTD effect below surface (adjusts curve of relationship)	5
δ	4	Maximum runoff at slope = 0 (i.e. δ - κ)	1.4
ε	5	Maximum runoff at slope = 0 (i.e. ε - κ)	1.46
η	5	Strength of WTD effect above surface (adjusts slope of relationship)	0.01
θ	5	Exponent of WTD effect above surface (adjusts curve of relationship)	50
κ	3,4,5	Minimum slope effect (i.e. when slope = 0)	0.5
λ	3,4,5	Strength of slope effect (adjusts how quickly runoff increases with slope)	4.5

Evaluating model performance

The model was run for three British blanket bogs for which observed WTD data were available (see Fig. 1 in main text). The sites were Moor House (northern England), Lake Vyrnwy (mid Wales) and Oughtershaw Moss (northern England); summaries of datasets are presented in the table below. Observed water tables were converted to monthly means for use in evaluation.

Sites differed in condition and monitoring method so datasets may vary in suitability for use in evaluation. As the model is parameterised for an intact peatland, it may not represent the hydrology at drained sites well. At Lake Vyrnwy, manual dipwell data were not frequent enough to represent true monthly means, so may not be well-predicted. Oughtershaw Moss data allow effects of peatland drainage to be explicitly examined, but as data span only 18 months, longer-term behaviours cannot be evaluated. Consequently, results should be interpreted in the context of site condition and monitoring method.

To generate model predictions, the model was driven with monthly climate data. Monthly predictions started in 1914, at the start of the UK Met Office 5 km gridded climate data[19]. All models were driven by these data until 2010, apart from the Moor House run, which was driven by data from a local ECN weather station from 1999 onwards; these data were gap-filled using the UK Met Office gridded data, which were first locally calibrated via regression against the ECN weather station data.

To evaluate model performance, measures of water table position (mean, maximum, minimum and standard deviation) were calculated for modelled and observed data. To examine how well fluctuations were replicated, modelled WTD was regressed against observed WTD. Finally, root mean squared error (RMSE) was calculated to examine model accuracy. All analyses were conducted in R v. 2.15 [20].

Summary of observed water table datasets used in model evaluation, indicating site location, peatland condition, and monitoring method.

Site	Location	Peatland drainage status	Number and type of dipwells	Duration of dipwell data	Dipwell recording frequency	Data source	Extra information
Moor House	Northern England (54° 41' N, 2° 22' W)	Intact peat	1 automatic dipwell	January 1999 – December 2010	Hourly	Environmental Change Network	Automatic dipwell readings calibrated against fortnightly manual dipwell readings to correct for systematic bias in automatic dipwell. Further site information in Heinemeyer *et al.* [1].
Lake Vyrnwy	Mid Wales (52° 47' N, 3° 35' W)	Drains blocked in 2007	24 manual dipwells	November 2007 – November 2010	Fortnightly or monthly	Active Blanket Bog Wales EU LIFE Project/RSPB	Three blocked drains crossed by a single dipwell transect; dipwells at distances of 0.5 – 10 m from the drains. Further information on site and data in Wilson *et al.* [21].
Oughtershaw Moss	Northern England (54° 13' N, 2° 14' W)	3 areas: one intact, one drained, one blocked in 1999	9 automatic dipwells in each area	January 2005 – June 2006	Every 20 minutes	Prof. Joseph Holden and Dr. Zoe Wallage, University of Leeds	One transect of 9 dipwells in each area; dipwells at distances of 1 – 34 m from drains in blocked and drained areas. Further information on site and data in Holden *et al.* [17].

Evaluation results

Modelled WTDs were compared to observed WTDs for all sites (see main text Fig. 2 and figure below). Model performance was best at sites where dipwells monitored intact peat, producing the best results in both WTD position and fluctuations. Fluctuations were reasonably well predicted even in drained sites, but WTD position became less well predicted. This is to be expected, as after drain blocking, local hydrology may reach an intermediate state between intact and drained[17, 21]. Further, the nature of sampling could have influenced evaluation results. Whilst Moor House and Oughtershaw Moss used automated dipwells, at Lake Vyrnwy dipwells were sampled manually once per month, meaning that WTDs did not represent true monthly means; such sampling effects could contribute significantly to the slightly weaker performance at Lake Vyrnwy.

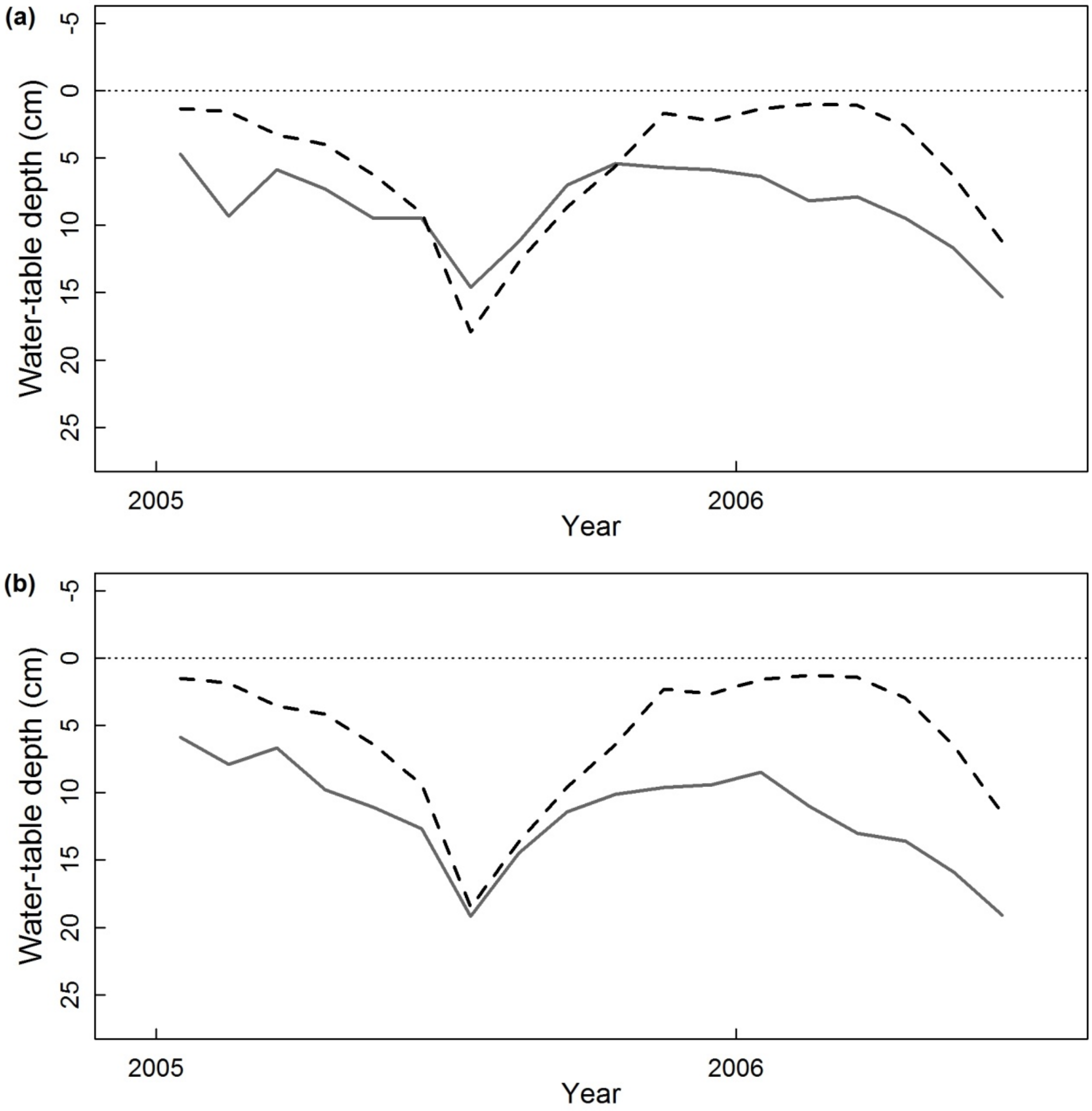

Time series of observed (grey, solid line) and modelled (black, dashed line) mean monthly WTD for Oughtershaw Moss, for a) peat with blocked drains, and b) drained peat. Dotted line indicates peat surface; positive WTD values indicate a water table below the surface; negative values indicate surface ponding.

Linear regressions indicated that modelled WTD was a highly significant predictor of observed WTD (see table below). Most variation was explained for the intact Oughtershaw Moss site (65.3%), although over 50% of variation was also explained at the other two Oughtershaw Moss sites and Moor House. Least variation (47.4%) was explained at Lake Vyrnwy.

Results from linear regressions of observed WTD vs. modelled WTD.

	Intercept (± SE)	Slope (± SE)	Test statistic	R^2
Moor House (I)	0.595 ± 0.350, $P = 0.091$	0.813 ± 0.060, $P < 0.001$	$F_{1,142} = 185.80$	0.567
Lake Vyrnwy (B)	-0.726 ± 0.630, $P = 0.258$	0.545 ± 0.107, $P < 0.001$	$F_{1,29} = 26.15$	0.474
Oughtershaw (I)	2.474 ± 0.804, $P = 0.007$	0.581 ± 0.106, $P < 0.001$	$F_{1,16} = 30.05$	0.653
Oughtershaw (D)	8.256 ± 0.956, $P < 0.001$	0.576 ± 0.126, $P < 0.001$	$F_{1,16} = 20.81$	0.565
Oughtershaw (B)	6.036 ± 0.762, $P < 0.001$	0.472 ± 0.106, $P < 0.001$	$F_{1,16} = 19.84$	0.554

The model predicted mean WTD to within 0.2 cm at Moor House and 0.1 cm at the intact Oughtershaw Moss site (see table below). At sites with blocked or open drainage ditches, WTD position was less well predicted, with an error in mean position of 2.2 cm at Lake Vyrnwy, 3.1 cm at the blocked Oughtershaw Moss site, and 5.7 cm at the drained Oughtershaw Moss site. WTD standard deviation was predicted to within 0.32 cm at Moor House, although at the all other sites the model predicted larger standard deviations than observed (see table below). Minimum water-table positions were predicted to within 1 – 2 cm for both Moor House and Oughtershaw Moss intact sites; RMSE was 2.9 cm for both of these sites. RMSE at Lake Vyrnwy was only 3.9 cm, and minimum WTD was predicted to within 2 cm. However, for Oughtershaw Moss blocked and drained sites, minimum WTD was only predicted to within 3 – 5 cm and RMSE was 4.5 cm and 6.6 cm respectively. Maximum WTD was predicted to within 1.8 cm at Moor House, and 0.7 cm at the Oughtershaw Moss drained site, but within only 2 – 5 cm elsewhere.

Mean, maximum and minimum monthly WTD values for observed data and for MILLENNIA model predictions. 'I' refers to intact peat; 'B' refers to peat with blocked drainage ditches; 'D' refers to peat with open drainage ditches.

	Mean WTD ± S.D. (cm)		Max WTD (cm)		Min WTD (cm)		RMSE (cm)
	Observed	Predicted	Observed	Predicted	Observed	Predicted	
Moor House (I)	4.1 ± 4.33	4.3 ± 4.01	22.9	21.1	-1.7	-0.5	2.9
Lake Vyrnwy (B)	1.8 ± 2.90	4.4 ± 3.46	8.7	13.5	-2.7	-0.7	3.9
Oughtershaw (I)	5.9 ± 3.58	5.8 ± 4.98	14.2	18.6	2.0	1.3	2.9
Oughtershaw (D)	11.6 ± 3.78	5.9 ± 4.93	19.2	18.5	5.9	1.3	6.6
Oughtershaw (B)	8.6 ± 3.05	5.5 ± 4.81	15.3	17.9	4.7	1.0	4.5

Evaluation statistics compare favourably with those of other peatland WTD models. A model of mined Canadian bogs[22] showed a difference between modelled and observed annual mean WTD of around 5 cm, and a difference in standard deviation of 0.2 – 0.3 cm. A model of Finnish bogs[23] showed a mean error across a year of 2.6 – 3.3 cm, with the maximum differences observed 11 – 13 cm. A hydrological model of a drained British bog[24] produced RMSE values of 2.7 – 16.3 cm. Therefore, values reported here are of a similar magnitude to those reported for other models.

Supplementary References

1. Heinemeyer A*, et al.* The MILLENNIA peat cohort model: predicting past, present and future soil carbon budgets and fluxes under changing climates in peatlands. *Clim Res* **45**, 207-226 (2010).

2. Clark JM*, et al.* Model inter-comparison between statistical and dynamic model assessments of the long-term stability of blanket peat in Great Britain (1940-2099). *Clim Res* **45**, 227-248 (2010).

3. Bauer IE. Modelling effects of litter quality and environment on peat accumulation over different time-scales. *Journal of Ecology* **92**, 661-674 (2004).

4. Brunsdon C, McClatchey J, Unwin DJ. Spatial variations in the average rainfall–altitude relationship in Great Britain: an approach using geographically weighted regression. *International Journal of Climatology* **21**, 455-466 (2001).

5. Lennon JJ, Turner JRG. Predicting the spatial distribution of climate: temperature in Great Britain. *J Anim Ecol* **64**, 370-392 (1995).

6. Bennie J, Huntley B, Wiltshire A, Hill MO, Baxter R. Slope, aspect and climate: spatially explicit and implicit models of topographic microclimate in chalk grassland. *Ecol Model* **216**, 47-59 (2008).

7. Thornthwaite CW. An approach toward a rational classification of climate. *Geographical Review* **38**, 55-94 (1948).

8. Dolan TJ, Hermann AJ, Bayley SE, Zoltek Jr J. Evapotranspiration of a Florida, U.S.A., freshwater wetland. *Journal of Hydrology* **74**, 355-371 (1984).

9. Brooks KN. Surface hydrology. In: *The Patterned Peatlands of Minnesota* (eds Wright HE, Coffin B, Aaseng NE). University of Minnesota Press (1992).

10. Brooks KN, Verma SB, Kim J, Verry ES. Scaling up evapotranspiration estimates from process studies to watersheds. In: *Peatland Biogeochemistry and Watershed Hydrology at the Marcell Experimental Forest* (eds Kolka RK, Sebestyen SD, Verry ES). CRC Press (2011).

11. Guertin DP, Barten PK, Brooks KN. The peatland hydrologic impact model: development and testing. *Nordic Hydrology* **18**, 79-100 (1987).

12. Nungesser MK. Modelling microtopography in boreal peatlands: hummocks and hollows. *Ecol Model* **165**, 175-207 (2003).

13. Koerselman W, Beltman B. Evapotranspiration from fens in relation to Penman's potential free water evaporation (E_0) and pan evaporation. *Aquatic Botany* **31**, 307-320 (1988).

14. Evans MG, Burt TP, Holden J, Adamson JK. Runoff generation and water table fluctuations in blanket peat: evidence from UK data spanning the dry summer of 1995. *Journal of Hydrology* **221**, 141-160 (1999).

15. Chaplot VAM, Le Bissonnais Y. Runoff features for interrill erosion at different rainfall intensities, slope lengths, and gradients in an agricultural loessial hillslope. *Soil Science Society of America Journal* **67**, 844-851 (2003).

16. Meyles E, Williams A, Ternan L, Dowd J. Runoff generation in relation to soil moisture patterns in a small Dartmoor catchment, Southwest England. *Hydrological Processes* **17**, 251-264 (2003).

17. Holden J, Wallage ZE, Lane SN, McDonald AT. Water table dynamics in undisturbed, drained and restored blanket peat. *Journal of Hydrology* **402**, 103-114 (2011).

18. ECN Data Centre. http://data.ecn.ac.uk accessed 25.06.12.

19. Perry M, Hollis D. The generation of monthly gridded datasets for a range of climatic variables over the UK. *International Journal of Climatology* **25**, 1041-1054 (2005).

20. R Development Core Team. R: A Language and Environment for Statistical Computing. R Foundation for Statistical Computing Vienna, Austria. http://www.R-project.org/ (2012)

21. Wilson L, Wilson J, Holden J, Johnstone I, Armstrong A, Morris M. Recovery of water tables in Welsh blanket bog after drain blocking: discharge rates, time scales and the influence of local conditions. *Journal of Hydrology* **391**, 377-386 (2010).

22. Kennedy GW, Price JS. Simulating soil water dynamics in a cutover bog. *Water Resources Research* **40**, W12410 (2004).

23. Weiss R, Shurpali NJ, Sallantaus T, Laiho R, Laine J, Alm J. Simulation of water table level and peat temperatures in boreal peatlands. *Ecol Model* **192**, 441-456 (2006).

24. Ballard CE, McIntyre N, Wheater HS, Holden J, Wallage ZE. Hydrological modelling of drained blanket peatland. *Journal of Hydrology* **407**, 81-93 (2011).
